# Autonomous materials discovery driven by Gaussian process regression with inhomogeneous measurement noise and anisotropic kernels

**DOI:** 10.1038/s41598-020-74394-1

**Published:** 2020-10-19

**Authors:** Marcus M. Noack, Gregory S. Doerk, Ruipeng Li, Jason K. Streit, Richard A. Vaia, Kevin G. Yager, Masafumi Fukuto

**Affiliations:** 1 The Center for Advanced Mathematics for Energy Research Applications (CAMERA), Lawrence Berkeley National Laboratory, Berkeley, CA 94720 USA; 2 Center for Functional Nanomaterials, Brookhaven National Laboratory, Upton, NY 11973 USA; 3 National Synchrotron Light Source II, Brookhaven National Laboratory, Upton, NY 11973 USA; 4 Materials and Manufacturing Directorate, Air Force Research Laboratories, Wright-Patterson Air Force Base, OH 45433 USA

**Keywords:** Chemistry, Energy science and technology, Engineering, Materials science, Mathematics and computing, Physics

## Abstract

A majority of experimental disciplines face the challenge of exploring large and high-dimensional parameter spaces in search of new scientific discoveries. Materials science is no exception; the wide variety of synthesis, processing, and environmental conditions that influence material properties gives rise to particularly vast parameter spaces. Recent advances have led to an increase in the efficiency of materials discovery by increasingly automating the exploration processes. Methods for autonomous experimentation have become more sophisticated recently, allowing for multi-dimensional parameter spaces to be explored efficiently and with minimal human intervention, thereby liberating the scientists to focus on interpretations and big-picture decisions. Gaussian process regression (GPR) techniques have emerged as the method of choice for steering many classes of experiments. We have recently demonstrated the positive impact of GPR-driven decision-making algorithms on autonomously-steered experiments at a synchrotron beamline. However, due to the complexity of the experiments, GPR often cannot be used in its most basic form, but rather has to be tuned to account for the special requirements of the experiments. Two requirements seem to be of particular importance, namely inhomogeneous measurement noise (input-dependent or non-i.i.d.) and anisotropic kernel functions, which are the two concepts that we tackle in this paper. Our synthetic and experimental tests demonstrate the importance of both concepts for experiments in materials science and the benefits that result from including them in the autonomous decision-making process.

## Introduction

Artificial intelligence and machine learning are transforming many areas of experimental science. While most techniques focus on analyzing “big data” sets, which are comprised of redundant information, i.e. information that is not strictly needed to define the model confidently, collecting smaller but information-rich data sets has become equally important. Brute-force data collection leads to tremendous inefficiencies in the utilization of experimental facilities and instruments, in data analysis and data storage; large experimental facilities around the globe are running at 10–20% utilization and are still spending millions of dollars each year to keep up with the increase in the amount of data storage needed^1–4^. In addition, conventional experiments require scientists to prepare samples and directly control experiments, which leads to highly-trained researchers spending significant effort on micromanaging experimental tasks rather than thinking about scientific meaning. To avoid this problem, autonomously steered experiments are emerging in many disciplines. These techniques place measurements only where they can contribute optimally to the overall knowledge gain. Measurements that collect redundant information are avoided. These autonomous approaches minimize the number of needed measurements to reach a certain model confidence, thus optimizing the utilization of experimental, computing, and data-storage facilities. Autonomy, in the course of this paper, refers to the machine’s ability to self-drive measurements of an experiment. Some initial parameters, such as the parameters to explore and their corresponding ranges, have to be defined by the user beforehand.

A universal goal in materials science is to explore the characteristics of a given material across the set of all conceivable combinations of experimental parameters, which can be thought of as a parameter space defining that class of materials. The experimental parameters can be the characteristics of material components, their composition, processing or synthesis parameters, and environmental conditions on which the experimental outcomes depend^5,6^. Successful exploration of the parameter space amounts to being able to define a high-confidence map, i.e. a surrogate model function, of experimental outcomes across all elements of the set. For two-dimensional parameter spaces, this is traditionally achieved by “scanning” the space, often on a simple Cartesian grid. Selecting a scanning strategy implies picking a scan resolution without knowing the model function, which will unequivocally lead to inaccuracies and inefficiencies. When the parameter space is high-dimensional, an approach based on intuition is often used, i.e., manually selecting measurements, assessing trends and patterns in the data, and selecting follow-up measurements. With increasing dimensionality of the parameter space, this method quickly fails to efficiently explore the space and becomes prone to bias. Needless to say, the human brain is generally poorly equipped for high-dimensional pattern recognition.

What is needed are methods that decouple the human from the measurement selection process. This fact served as a motivation to establish a research field called design of experiment (DOE)^7^, which can be traced back as far as the late 1800s. These DOE methods are largely geometrical, independent of the measurement outcomes, and are concerned with efficiently exploring the entire parameter space. The latin-hyper-cube method is the prime example of this class of methods^8,9^. Most of the recent approaches to steer experiments are part of a field called active learning, which is based on machine learning techniques^5,10–12^. Others have used deep neural networks to make data acquisition cheaper^13^. Many techniques originated from image analysis^11,14^, but, as images are traditionally two or three dimensional, these methods rarely scale efficiently to high-dimensional spaces. A useful collection of methods can be found in^15,16^.

Gaussian process regression (GPR) is a particularly successful technique to steer experiments autonomously^17,18^. The success of GPR in steering experiments is due to its non-parametric nature; simply speaking, the more data that is gathered the more complicated the model function can become. The number of parameters of the function, and therefore its complexity, does not have to be defined a priori. This is in contrast to neural networks, which need a specification of an architecture (number of layers, layer width, activation function) beforehand. The non-parametric nature is not unique to Gaussian processes, but is characteristic to all kernel methods and, in an even broader scope, all methods that approximate a function by a sum of basis functions. The strength of Gaussian processes comes from the fact that kernels are used to define a similarity measure between points, which in turn is used to define a covariance matrix. Therefore, GPR also naturally includes uncertainty quantification, which is an absolute necessity in experimental sciences.

Traditional GPR has mostly been derived and applied under the assumption of independent and identically distributed noise (i.i.d. noise)^18–23^, i.e., noise that follows the same probability density function at each measurement point. Since we are exclusively dealing with Gaussian statistics, this means that all measurements have the same variance. In Kriging, the geo-statistical analog of GPR, this concept is called the nugget effect, named after gold nuggets in the sub-surface. In early geo-statistical computations, the gold nuggets lead to seemingly random errors. These were assumed to be constant across the domain. However, for materials-discovery experiments the assumption of i.i.d. noise is an unacceptable simplification. The variance of real experimental measurements vary greatly across the parameter space, and this has to be reflected in the steering process as well as in the final model creation. For instance, in x-ray scattering experiments, the variance of a raw measurement depends strongly on the exposure time; computed quantities can have wildly different variances depending on the raw data in that part of the space (e.g. fit quality will not be uniform), and material heterogeneity will depend strongly on location within the parameter space. These inhomogeneities in the measurement noise need to be actively included in the final model to avoid interpolation mistakes and consequently erroneous models. Fortunately, non-i.i.d. noise can easily be included in the GPR framework^24,25^. Large variances have to be countered with more measurements in the respective areas until the desired uncertainty threshold is reached. This is naturally taken care of by the non-i.i.d Gaussian process since the overall posterior variance (or prediction variance) is a combination of the measurement variance and the variance due to distances from known data. When creating the final model, the algorithm has to incorporate that the final model function does not have to explain data points exactly if there is an associated variance. Therefore, the model function does not have to pass through every data point. After correct tuning, GPR is perfectly equipped for this situation since it keeps track of a probability distribution over all possible model functions; conditioning will then produce the most likely model function incorporating all measurement variances optimally.

**Figure 1:**
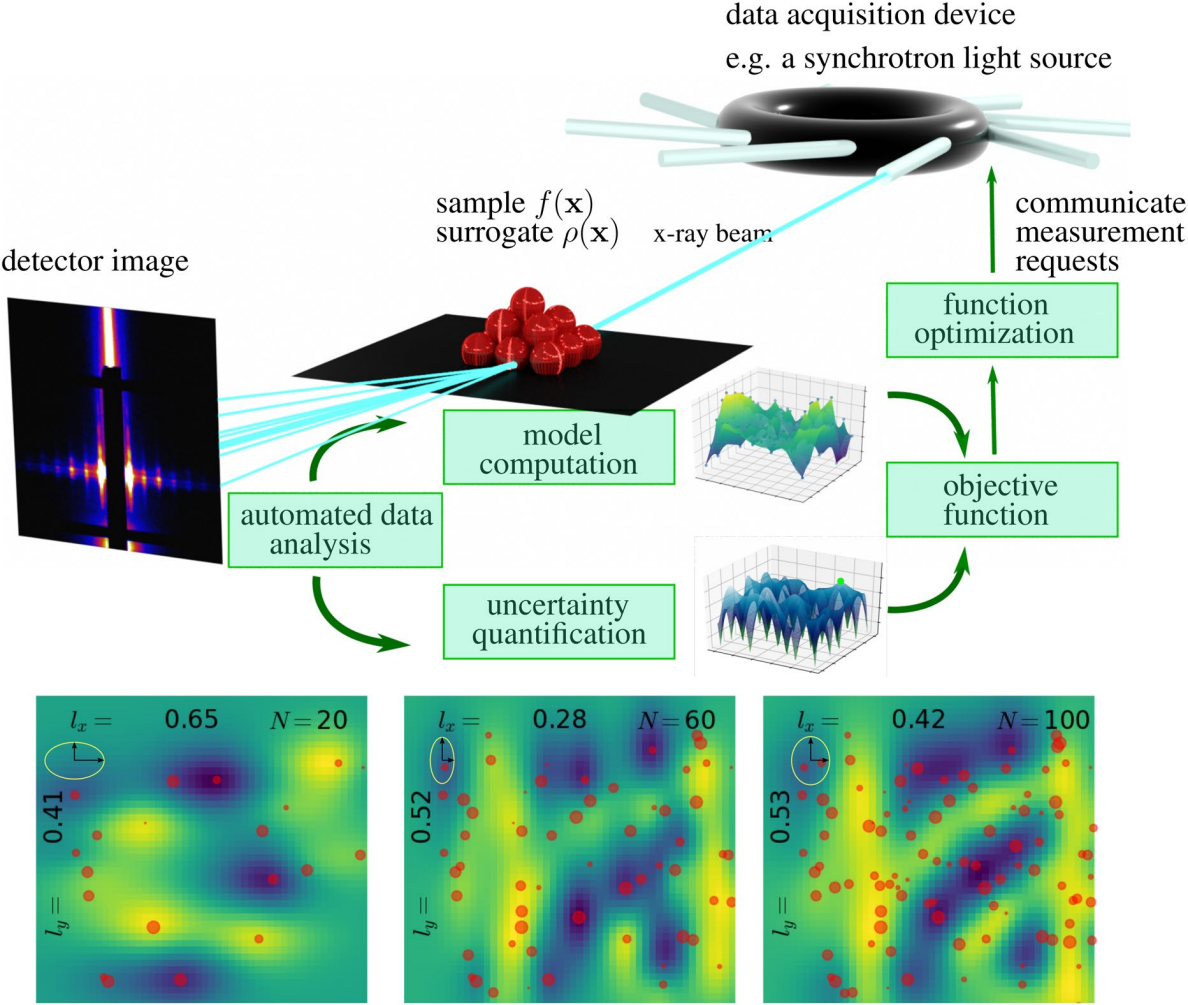
Schematic of an autonomous experiment. The data acquisition device in this example is a beamline at a synchrotron light source. The measurement result depends on parameters $\mathbf{x}$. The raw data is then sent through an automated data processing and analysis pipeline. From the analyzed data, the autonomous-experiment algorithm creates a surrogate model and an uncertainty function whose maxima represent points of high-value measurements; they are found by employing function optimization tools. The new measurement parameters $\mathbf{x}$ are then communicated to the data acquisition device and the loop starts over. The main contribution of the present work is that the model computation and uncertainty quantification account for the anisotropic nature of the model function and the input-dependent (non-i.i.d.) measurement noise. The surrogate model (bottom) shows how the model function is evolving as the experiment is steered and more data (*N*) is collected. The red dots indicate the positions of the measurements and their size represents the varying associated measurement variances. The numbers $l_x$ and $l_y$ indicate the anisotropic correlation lengths that the algorithm finds by maximizing a log-likelihood function. The ellipses show the found anisotropy visually. The take-home message for the practitioner here is that the method will find the most likely model function given all collected data with their variances. The model function will not pass directly through the points but find the most likely shape given all available information.

Another effect that has a significant impact on autonomous experiments is anisotropy of the parameter space, which is either introduced by differing parameter ranges or different model variability in different parameter-space directions. In isotropic GPR one finds a single characteristic length scale for the data set. This was again motivated by early geo-statistical surveys in which isotropy was a good assumption. However, when one of the parameters is of significantly different magnitude, for instance, spatial directions in $\text {mm}~\in ~[0,1]$ versus temperature in $^\circ \mathrm {C}~\in ~[5,500]$, we should find different length scales for different directions of the parameter space. Also, there might be different differentiability characteristics in different directions. It is therefore vitally important to give the model the flexibility to account for those varying features. This can either be done by using an altered Euclidean norm, or by employing different norms that provide more flexibility of distance measures in different directions. The general idea, including the concepts proposed in this paper, is visualized in Fig. 1.

The proposed method can be understood as a variant of Bayesian optimization (BO) in which only Gaussian priors and likelihoods are considered. While, as the name suggests, BO is mostly used to find a maximum or minimum, autonomous experimentation makes no such restriction. However, since there is a variety of different objective functions that can be optimized in BO, the proposed method can certainly be understood as a subset of BO. See^26^ for a good overview of Bayesian optimization.

This paper is organized as follows: First, we introduce the traditional theory of Gaussian process regression with i.i.d. noise and standard isotropic kernel functions. Second, we make formal changes to the theory to include non-i.i.d. noise and anisotropy. Third, we demonstrate the impact of the two concepts on synthetic experiments. Fourth, we present a synchrotron beamline experiment that exploited both concepts for autonomous control.

## Gaussian process regression with non-i.i.d. noise and anisotropic kernels

### Prerequisite

**Figure 2:**
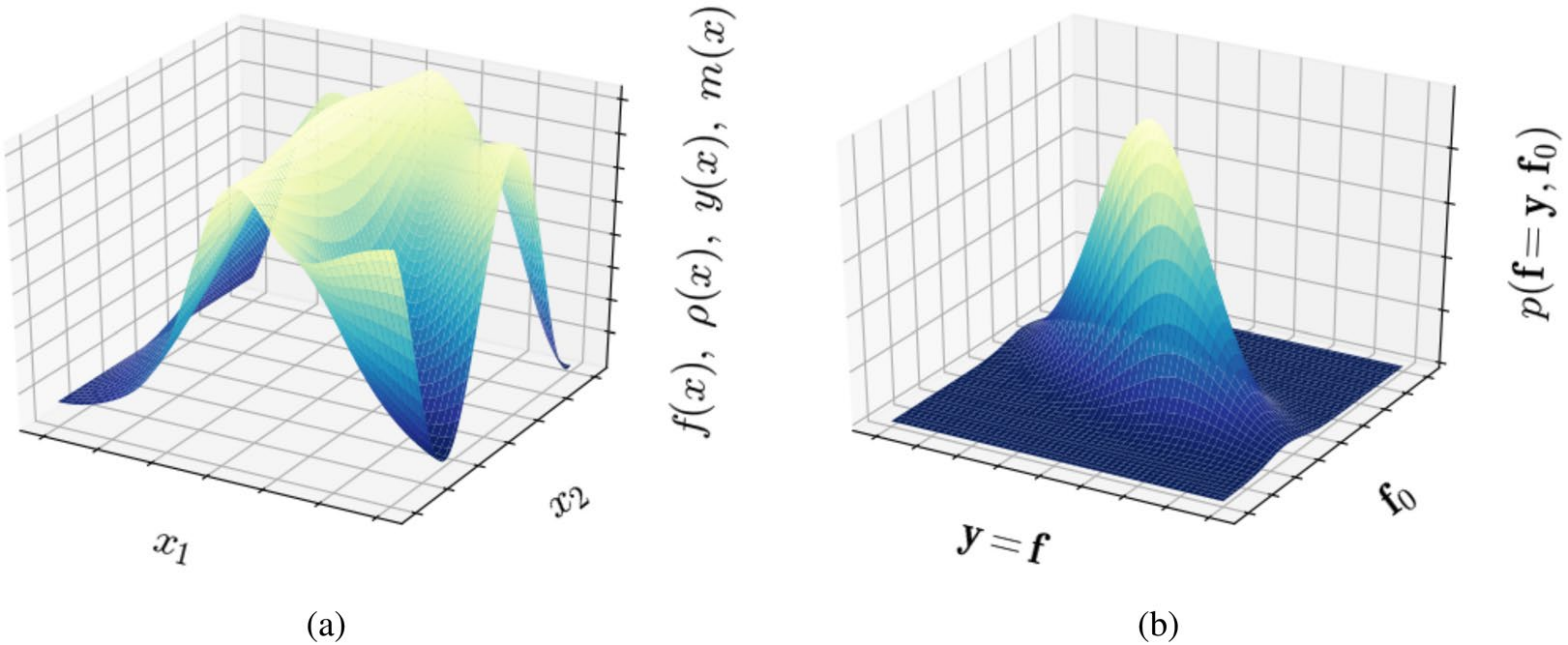
Figure emphasizing the distinction between the spaces and functions involved in the derivation. (**a**) A function over $\mathscr {X}$. This can be the surrogate model $\rho (\mathbf{x})$, the latent function $f(\mathbf{x})$ to be approximated through an experiment, the function describing the measurements $y(\mathbf{x})$ or the predictive mean function $m(\mathbf{x})$. $x_1$ and $x_2$ are two experimentally controlled parameters (e.g., synthesis, processing or environmental conditions) that the measurement outcomes potentially depend on. (**b**) The Gaussian probability density function over $\mathscr {H}$ which gives GPR its name. For noise-free measurements, $\mathbf{y}~=~\mathbf{f}$ at measurement points, meaning that we can directly observe the model function. Generally this is not the case and the observations $\mathbf{y}$ are corrupted by input-dependent (non-i.i.d) noise.

We define the parameter space ${\mathscr{X}} \subset {\mathbb{R}}^n$, which serves as the index set or input space in the scope of Gaussian process regression and elements ${\mathbf{x}}~\in {\mathscr{X}}$. We define four functions over ${\mathscr {X}}$. First, the latent function $f=f({\mathbf{x}})$ can be interpreted as the inaccessible ground truth. Second, the often noisy measurements are described by $y=y({\mathbf{x}}):\; {\mathscr {X}}\; \rightarrow \, {\mathbb {R}}^d$. To simplify the derivation, we assume $d=1$; allowing for $d>1$ is a straightforward extension. Third, the surrogate model function is then defined as $\rho =\rho ({\mathbf{x}}):~{\mathscr {X}}~\rightarrow ~{\mathbb {R}}$. Fourth, the posterior mean function $m({\mathbf{x}})$, which is often assumed to equal the surrogate model, i.e., $m({\mathbf{x}})=\rho ({\mathbf{x}})$, but this is not necessarily the case. We also define a second space, a Hilbert space ${\mathscr {H}}~\subset ~{\mathbb {R}}^N~\times ~{\mathbb {R}}^N~\times ~{\mathbb {R}}^J$, with elements $[{\mathbf{f}}~{\mathbf{y}}~{\mathbf{f}}_0]^T$, where *N* is the number of data points, *J* is the number of points at which we want to predict the model function value, ${\mathbf{y}}$ are the measurement values, ${\mathbf{f}}$ is the vector of unknown latent function evaluations and ${\mathbf{f}}_0$ is the vector of predicted function values at a set of positions. Note that scalar functions over ${\mathscr {X}}$, e.g. $f({\mathbf{x}})$, are vectors (bold typeface) in the Hilbert space ${\mathscr {H}}$, e.g. ${\mathbf{f}}$. We also define a function *p* over our Hilbert space which is just the function value of the Gaussian probability density functions involved. For more explanation on the distinction between the two spaces and the functions involved see Fig. 2.

### Gaussian process regression with isotropic kernels and i.i.d. observation noise

Defining a GP regression model from data $D=\{({\mathbf{x}}_1,y_1),\ldots ,({\mathbf{x}}_N,y_N)\}$, where $y_i=f({\mathbf{x}}_i)+\epsilon ({\mathbf{x}}_i)$, is accomplished in a GP regression framework, by defining a Gaussian probability density function, called the prior, as

1 $$\begin{aligned} p({\mathbf{f}})=\frac{1}{\sqrt{(2\pi )^{\mathrm {dim}}|{\mathbf{K}}|}} \exp \left[ -\frac{1}{2}({\mathbf{f}}-{\varvec{\mu }})^T {\mathbf{K}}^{-1}({\mathbf{f}}-\varvec{\mu }) \right] , \end{aligned}$$

and a likelihood

2 $$\begin{aligned} p({\mathbf{y}})=\frac{1}{\sqrt{(2\pi )^{\mathrm {dim}}}\sigma } \exp \left[ -\frac{1}{2\sigma ^2}({\mathbf{y}} - {\mathbf{f}})^T ({\mathbf{y}} - {\mathbf{f}}) \right] , \end{aligned}$$

where $\varvec{\mu }=[\mu ({\mathbf{x}}_1),\ldots ,\mu ({\mathbf{x}}_N)]^T$ is the mean of the prior Gaussian probability density function (not to be confused with the posterior mean function $m({\mathbf{x}})$). Here *dim* is the dimensionality of the space over which the Gaussian probability density function is defined. The prior mean can be understood as the position of the Gaussian. ${\mathbf{f}}=[f({\mathbf{x}}_1),\ldots ,f({\mathbf{x}}_N)]^T$, ${\mathbf{K}}_{ij}~=~{\mathbf{k}}(\phi ,{\mathbf{x}}_i,{\mathbf{x}}_j);~{\mathbf{x}}\in {\mathscr {X}}$ is the covariance of the Gaussian process, with its covariance function, often referred to as the kernel, $k(\phi ,{\mathbf{x}}_i,{\mathbf{x}}_j)$, where $\phi$ is a set of hyper parameters, most often length scales and signal variance, and where $\sigma ^2$ is the variance of the i.i.d. observation noise. The hyper parameters will be later often referred to as length scale *l* or signal variance $\sigma _s^2$. We will omit the dependency on $\phi$ in the kernel definition unless necessary for clarity. The problem here is that, in practice, the i.i.d. noise restriction rarely holds in experimental sciences, which is one of the issues to be addressed in this paper. The kernel *k* is a symmetric and positive semi-definite function, such that $k:{\mathscr {X}} \times {\mathscr {X}}\rightarrow {\mathbb {R}}$. As a reminder, ${\mathscr {X}}$ is our parameter space and often referred to as index set or input space in the literature. A well-known choice^19^ is the Matérn kernel class defined by

3 $$\begin{aligned} k({\mathbf{x}}_i,{\mathbf{x}}_j)~=~ \sigma _s^2 \frac{2^{(1-\nu )}}{\Gamma (\nu )} \Bigg ( \sqrt{2\nu }\frac{r}{l }\Bigg )^{\nu }B_{\nu }\Bigg (\sqrt{2\nu }{\frac{r}{l}}\Bigg ), \end{aligned}$$

where $B_\nu$ is the Bessel function of second kind, $\Gamma$ is the gamma function, $\sigma _s^2$ is the signal variance, *l* is the length scale, $r~=~||{\mathbf{x}}_i-{\mathbf{x}}_j||_{l_2}$ is the Euclidean distance between input points and $\nu$ is a parameter that controls the differentiability characteristics of the kernel and therefore of the final model function. The well-known exponential and squared exponential kernels are special cases of the Matérn kernels for $\nu ~=~\frac{1}{2}$ and $\nu ~\rightarrow ~\infty$ respectively. Unless otherwise stated, we used the Matérn kernel with $\nu ~=~\frac{3}{2}$ for our tests and experiments, which translates to first order differentiability of the posterior mean function. The signal variance $\sigma _s^2$ and the length scale *l* are hyper parameters ($\phi$) that are found by maximizing the log-likelihood, i.e., solving

4 $$\begin{aligned} \mathop {\hbox {arg max}}\limits _{\phi ~\mu }\Big (\log (L(D;\phi ,\mu ({\mathbf{x}}))) \Big ) \end{aligned}$$

where

5 $$\begin{aligned} \log (L(D;\phi ,\mu ({\mathbf{x}})))~=&~-\frac{1}{2}({\mathbf{y}}-\varvec{\mu })({\mathbf{K}}(\phi )+\sigma ^2~{\mathbf{I}})^{-1}({\mathbf{y}}-\varvec{\mu }) \nonumber \\&~-\frac{1}{2}\log (|{\mathbf{K}}(\phi )+\sigma ^2~{\mathbf{I}}|) -\frac{\dim }{2}\log (2\pi ), \end{aligned}$$

where *I* is the identity matrix. In the isotropic case, we only have to optimize for one signal variance and one length scale (per kernel function). The mean function $\mu ({\mathbf{x}})$, while formally being part of the optimization problem in (4) is often assumed to be constant and therefore neglected. The mean function assigns the location of the prior in ${\mathscr {H}}$ to any ${\mathbf{x}}~\in ~{\mathscr {X}}$; it can therefore be used to communicate prior knowledge (for instance physics knowledge) to the Gaussian process. For our tests and the experiment, we assume a constant mean function defined by the mean of the data. Choosing a particular kernel function and optimizing the hyper parameters can be a challenging task depending on the data and the function to be approximated. The kernel function has a dramatic impact on the approximation quality. It takes some practice and good knowledge of the characteristics of kernel functions and their effect on the Gaussian process to make the right decision. Provided some hyper parameters, the joint prior is given as

6 $$\begin{aligned} p({\mathbf{f}},{\mathbf{f}}_0)=\frac{1}{\sqrt{(2\pi )^{\mathrm {dim}}|{\varvec{\Sigma }}|}} \exp \left[ -\frac{1}{2} \Big ( \begin{bmatrix} {\mathbf{f}}-\varvec{\mu } \\ {\mathbf{f}}_0-\varvec{\mu }_0 \end{bmatrix}^T {\varvec{\Sigma }}^{-1} \begin{bmatrix} {\mathbf{f}}-\varvec{\mu } \\ {\mathbf{f}}_0-\varvec{\mu }_0 \end{bmatrix} \Big )\right] , \end{aligned}$$

where

7 $$\begin{aligned} \varvec{\Sigma }~=~ \begin{pmatrix} {\mathbf{K}} &{} \varvec{\kappa } \\ \varvec{\kappa }^T &{} \varvec{{\mathscr {K}}} \end{pmatrix}, \end{aligned}$$

where $\varvec{\kappa }_i~=~k(\phi ,{\mathbf{x}}_0,{\mathbf{x}}_i)$, $\varvec{{\mathscr {K}}}~=~k(\phi ,{\mathbf{x}}_0,{\mathbf{x}}_0)$ and, as a reminder, ${\mathbf{K}}_{ij}~=~k(\phi ,{\mathbf{x}}_i,{\mathbf{x}}_j)$. *dim* in (5) and (6) is again the dimensionality of the space the Gaussian probability density function is defined over. Intuitively speaking, $\varvec{\Sigma }$, ${\mathbf{K}}$ and *k* are all measures of similarity between measurement results $y({\mathbf{x}})$ of the input space. While $y({\mathbf{K}})$ stores this similarity between all data points, $\varvec{\Sigma }$ stores the similarity between all data points and all unknown points of interest, and $\varvec{{\mathscr {K}}}$ contains the similarity only between the unknown $y({\mathbf{x}})$ of interest. *k* contains the instruction on how to calculate this similarity. The reader might wonder: “How do we find the similarity between unknown points of interest?” The answer lies in the formulation of the kernels that calculate the similarity just by knowing locations ${\mathbf{x}}~\in ~{\mathscr {X}}$ and not the function evaluations $y({\mathbf{x}})$. ${\mathbf{x}}_0$ is the point where we want to estimate the mean and the variance. Note here that, with only slight adaption of the equation, we are able to compute the posterior mean and variance for several points of interest.

The predictive distribution is defined as

8 $$\begin{aligned} p({\mathbf{f}}_0|{\mathbf{y}})~&=~\int _{\mathbb{R}}^{\mathbb{N}} p({\mathbf{f}}_0|{\mathbf{f}},{\mathbf{y}})~p({\mathbf{f}},{\mathbf{y}})~d{\mathbf{f}} \nonumber \\&~\propto {\mathscr{N}}(\boldsymbol{\mu }+{\boldsymbol{\kappa }}^T~ ({\mathbf{K}}+\sigma ^2~{\mathbf{I}})^{-1}~({\mathbf{y}}-{\boldsymbol{\mu }}), {{\mathscr{K}}} - {\boldsymbol{\kappa }}^T~({\mathbf{K}}+\sigma ^2~{\mathbf{I}})^{-1}~{\boldsymbol{\kappa }}) \end{aligned}$$

and the predictive mean and the predictive variance are therefore respectively defined as

9 $$\begin{aligned} m({\mathbf{x}}_0)&=\varvec{\mu } + {\mathbf{k}}^T({\mathbf{K}}+\sigma ^2~{\mathbf{I}})^{-1}({\mathbf{y}}-\varvec{\mu }) \end{aligned}$$

10 $$\begin{aligned} \sigma ^2({\mathbf{x}}_0)&=k({\mathbf{x}}_0,{\mathbf{x}}_0)- {\mathbf{k}}^T({\mathbf{K}} + \sigma ^2~{\mathbf{I}})^{-1} {\mathbf{k}}, \end{aligned}$$

which are the posterior mean and variance at ${\mathbf{x}}_0$, respectively. ${\mathscr{N}}(\cdot ,\cdot )$ stands for the normal (Gaussian) distribution with a given mean and covariance.

### Gaussian processes with non-i.i.d. observation noise

To incorporate non-i.i.d. observation noise^27,28^ one can redefine the likelihood (2) as

11 $$\begin{aligned} p({\mathbf{y}})=\frac{1}{\sqrt{(2\pi )^{\dim }|{\mathbf{V}}|}} \exp \left[ -\frac{1}{2}({\mathbf{y}}-{\mathbf{f}})^T {\mathbf{V}}^{-1}({\mathbf{y}}-{\mathbf{f}}) \right] , \end{aligned}$$

where ${\mathbf{V}}$ is a diagonal matrix containing the respective measurement variances. In case the measurements happen to be correlated, the matrix ${\mathbf{V}}$ also has non-diagonal entries. However in our case this would have to be communicated by the instrument since we are not estimating the noise levels or their correlations^29^. We will only discuss and use non-correlated measurement noise in this paper.

From Eqs. (6) and (11), we can calculate Eq. (8), i.e., the predictive probability distribution for a measurement outcome at ${\mathbf{x}}_0$, given the data set. The mean and variance of this distribution are

12 $$\begin{aligned} m({\mathbf{x}}_0)&=\varvec{\mu } + {\mathbf{k}}^T({\mathbf{K}}+{\mathbf{V}})^{-1}({\mathbf{y}}-\varvec{\mu }) \end{aligned}$$

13 $$\begin{aligned} \sigma ^2({\mathbf{x}}_0)&=k(\mathbf{x_0},\mathbf{x_0})- {\mathbf{k}}^T({\mathbf{K}} + {\mathbf{V}})^{-1} {\mathbf{k}}, \end{aligned}$$

respectively. Note here, that the matrix of the measurement errors ${\mathbf{V}}$ replaces the matrix $\sigma ^2~{\mathbf{I}}$ in Eqs. (9) and (10). However, this does not follow from a simple substitution, but from a significantly different derivation. The log-likelihood (5) changes accordingly, yielding

14 $$\begin{aligned} \log (L(D;\varvec{\phi },\mu ({\mathbf{x}})))&= -\frac{1}{2}({\mathbf{y}}-\varvec{\mu })({\mathbf{K}}(\phi )+{\mathbf{V}})^{-1}({\mathbf{y}}-\varvec{\mu })\nonumber \\&\quad -\frac{1}{2}\log (|{\mathbf{K}}(\phi )+{\mathbf{V}}|) -\frac{\dim }{2}\log (2\pi ). \end{aligned}$$

**Figure 3:**
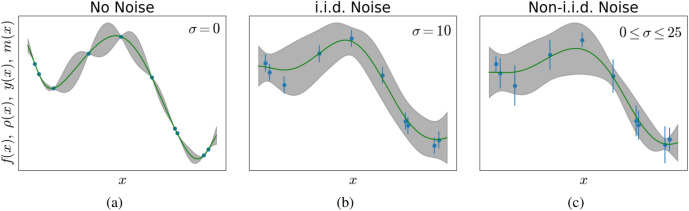
Three one-dimensional examples with (**a**) no noise, (**b**) i.i.d. noise and (**c**) non-i.i.d. noise, respectively. For the no-noise case, the model has to explain the data exactly. In the i.i.d. noise-case, the algorithm is free to choose a model that does not explain the data exactly but allows for a constant measurement variance. In the non-i.i.d. noise case, the algorithm finds the most likely model given varying variances across the data set. Note the vertical axis labels; *y*(*x*) are the measurement outcomes, *m*(*x*) is the mean function, i.e., the most likely model, $\rho (x)$ is the surrogate model, often assumed to equal the mean function and *f*(*x*) is the “ground truth” latent function.

This concludes the derivation of GPR with non-i.i.d. observation noise. Figure 3 illustrates the effect of different kinds of noise on an one-dimensional model function. As we can see, while some details of the derivation change when we account for inhomogeneous (also known as input-dependent or non-i.i.d) noise, the resulting equation are very similar and the computation exhibits no extra costs.

### Gaussian processes with anisotropy

**Figure 4:**
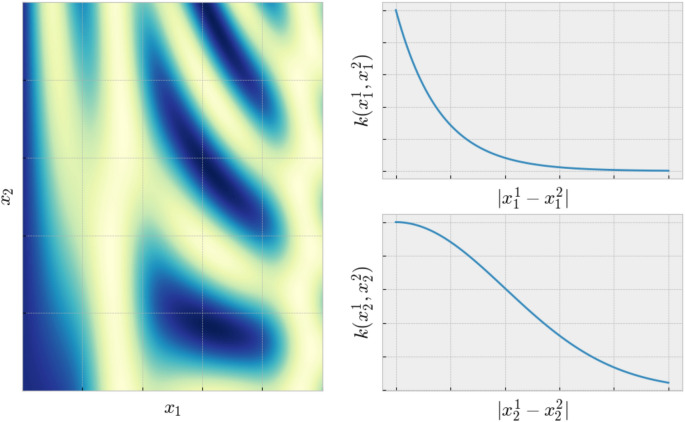
Model function with different length scales and different orders of differentiability in different directions. In $x_1$ direction we have assumed that the model function is not differentiable. Therefore we used the exponential kernel. In $x_2$ direction, the model can be differentiated an infinite number of times. We therefore chose the squared exponential kernel. For other orders of differentiability, other kernels can be used. Fixing the order of differentiability also gives the user the ability to incorporate domain knowledge into the experiment.

For parameter spaces ${\mathscr {X}}$ that are anisotropic, i.e., where different directions have different characteristic correlation length, we can redefine the kernel function to incorporate different length scales in different directions. One way of doing this for axial anisotropy is by choosing the $l^1$ norm as distance measure and redefine the kernel function as

15 $$\begin{aligned} k({\mathbf{x}}^m,{\mathbf{x}}^n) = \sigma _s^2~\prod _i^d~k_i(x^m_i~-~x^n_i;\phi _i), \end{aligned}$$

where the superscripts *m*, *n* mean point labels, the subscript *i* means different directions in ${\mathscr {X}}$ and *d* is here the dimensionality of ${\mathscr {X}}$. This kernel definition originates from the fact that multiplying kernels will result in another valid kernel^19^. Defining a kernel per direction gives us the flexibility to enforce different orders of differentiability in different directions of ${\mathscr {X}}$. The main benefit, however, is the possibility to define different length scales in different directions of ${\mathscr {X}}$ (see Fig. 4). Unfortunately, the choice of the $l^1$ norm can lead to a very recognizable checkerboard pattern in the surrogate model, but the predictive power of the associated variance function is significantly improved compared to the isotropic case.

A second way, which avoids the checkerboard pattern in the model but does not allow different kernels in different direction, is to redefine the distances in ${\mathscr {X}}$ as

16 $$\begin{aligned} r~=~\sqrt{{\mathbf{x}}^T~{\mathbf{M}}~{\mathbf{x}}}, \end{aligned}$$

where ${\mathbf{M}}$ is any symmetric positive semi-definite matrix playing the role of a metric tensor^30^. This is just the Euclidean distance in a transformed metric space. In the actual kernel functions, any *r*/*l* can then be replaced by the new equation for the metric. We will here only consider axis-aligned anisotropy, which means the matrix ${\mathbf{M}}$ is a diagonal matrix with the inverse of the length scales on its diagonal. The extension to general forms of anisotropy is straightforward but needs a more costly likelihood optimization since more hyper parameters have to be found. The rest of the theoretical treatment, however, remains unchanged. The mean function $\mu ({\mathbf{x}})$ and the hyper parameters $\phi$ are again found by maximizing the marginal log-likelihood (14). The associated optimization tries to find a maximum of a function that is defined over ${\mathbb {R}}^{d + 1}$, if we ignore the mean function as it is commonly done. We therefore have to find $d + 1$ parameters which adds a significant computational cost. If ${\mathbf{M}}$ is not diagonal we have to maximize the log-likelihood over ${\mathbb {R}}^{(d^2-N)/2 + 1}$. However, the optimization can be performed in parallel to computing the posterior variance, which can hide the computational effort. It is important to note that accounting for anisotropy can make the training of the algorithm, i.e. the optimization of the log-likelihood, significantly more costly. The extent of this depends on the kind of anisotropy considered. As we shall see, taking anisotropy into account leads to more efficient steering and a higher-quality final result, and is thus generally worth the additional computational cost.

## Synthetic tests

Our synthetic tests are carefully chosen to demonstrate the benefits of the two concepts under discussion, namely: non-i.i.d. observation noise and anisotropic kernels. To demonstrate the importance of including non-i.i.d. observation noise into the analysis, we consider a synthetic test based on actual physics which we used in previous work to showcase the functionality of past algorithms^17^. We are choosing an example given in a closed form because it provides a noise-free “ground truth” that we can compare to, whereas experimental data would inevitably include unknown errors. To showcase the importance of anisotropic kernels as part of the analysis, we provide a high-dimensional example based on a simulation of a material that is subject to a varying thermal history.

The shown synthetic tests explore spaces of very different dimensionality. There is no theoretical limit to the dimensionality of the parameter space. Indeed the autonomous methods described herein are most advantageous when operating in high-dimensional spaces since this is where simpler methods—and human intuition—typically fail to yield meaningful searches. However, while there is no theoretical limit, there are several practical issues that must be considered for high-dimensional problems. The quality of the approximation suffers in high-dimensional spaces since data density grows increasingly sparse with increasing dimensions. Therefore, in high-dimensional spaces often more data has to be gathered, which correspondingly increases computational costs. See^31–35^ for an overview of work on methods to speed up Gaussian process computations.

### Non-i.i.d. observation noise

For this test, we define a physical “ground truth” model $f({\mathbf{x}})$, whose correct function value at ${\mathbf{x}}$ would normally be inaccessible due to non-i.i.d measurement noise, but can be probed by our simulated experiment through $y({\mathbf{x}})$. In this case, we assume that the measurements are subject to Gaussian noise with a standard deviation of $2\%$ of the function value at ${\mathbf{x}}$. The ground-truth model function is defined to be the diffusion coefficient $D = D(r,T,C_m)$ for the Brownian motion of nanoparticles in a viscous liquid consisting of a binary mixture of water and glycerol:

17 $$\begin{aligned} D = \frac{k_B~T}{6\pi \mu r}, \end{aligned}$$

where $k_B$ is Bolzmann’s constant, $r~\in ~[1~,100]~\mathrm {nm}$ is the nanoparticle radius, $T~\in ~[0~,100]~^{\circ }\mathrm {C}$ is the temperature and $\mu =\mu (T,C_m)$ is the viscosity as given by^36^, where $C_m~\in ~[0.0,100.0]~\%$ is the glycerol mass fraction. This model was used in^17^ to show the functionality of Kriging based autonomous experiments. The experiment device has no direct access to the ground truth model, but adds an unavoidable noise level, i.e.,

18 $$\begin{aligned} D = \frac{k_B~T}{6\pi \mu r}~+~\epsilon (T,C_{m},r), \end{aligned}$$

**Figure 5:**
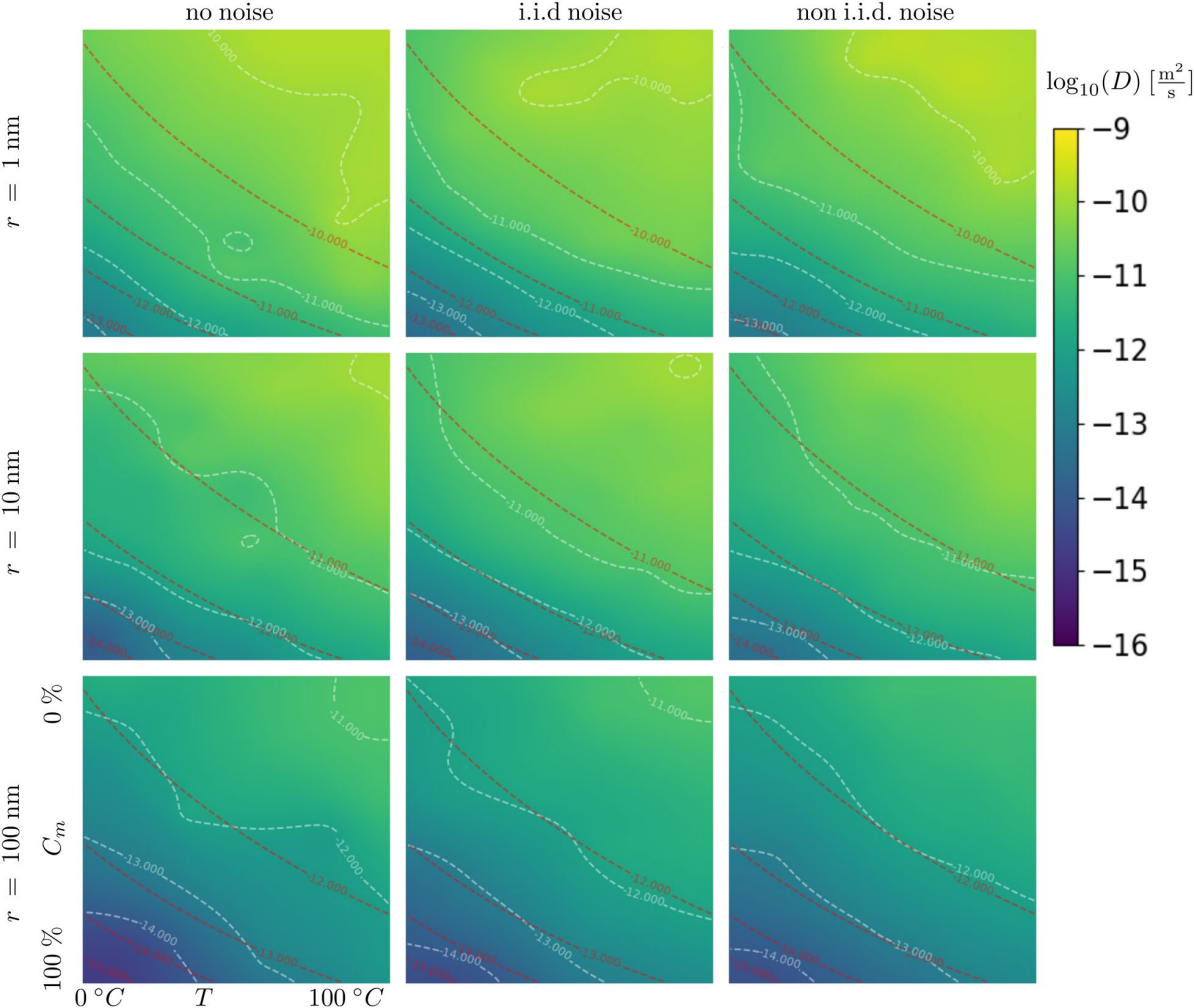
The result of the diffusion-coefficient example on a three-dimensional input space. The figure shows the result of the GP approximation after 500 measurements for three different nanoparticle radii. While the measurement results are always subject to differing noise, the model can take noise into account in different ways. Most commonly noise is ignored (left column). If noise is included, it is common to approximate it by i.i.d. noise (middle column). The proposed method models the noise as what it is, which is non-i.i.d. noise (right column). The iso-lines of the approximation are shown in white while the iso-lines of the ground truth are shown in red. Observe how the no-noise and the i.i.d. noise approximations create localized artifacts. The non-i.i.d. approximation does a far better job of creating a smooth model that explains all data including noise.

**Figure 6:**
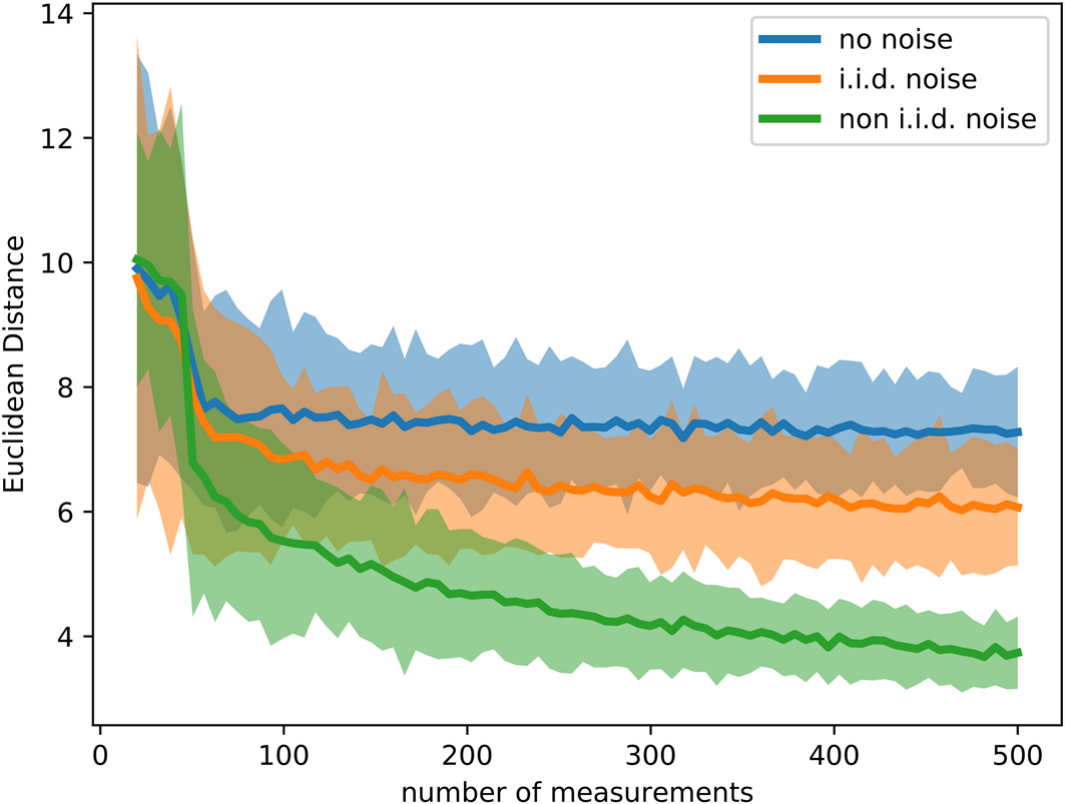
The approximation errors of the surrogate model during the diffusion-coefficient example (Fig. 5), for three different noise models noted in the legend. The bands around each line represent the standard deviation of this error metric computed by running repeated synthetic experiments.

To demonstrate the importance of the noise model, we first ignore the noise $\epsilon$, then approximate it assuming i.i.d. noise, and finally model it allowing for non-i.i.d. noise. Figure 5 shows the results after 500 measurements, and a comparison to the (inaccessible) ground truth. Figure 6 compares the decrease in the error, in form of the Euclidean distance between the models and the ground truth, with increasing number of measurements *N*, for the three different types of noise.

The results show that treating noise as i.i.d. or even non-existent can lead to artifacts in the surrogate model. Additionally, the discrepancy between the ground truth and the surrogate mode is reduced far more efficiently if non-i.i.d. noise is accounted for.

### Anisotropy

Allowing anisotropy can increase the efficiency of autonomous experiments significantly for any dimensionality of the underlying parameter space. However, as the dimensionality of the parameter space increases, the importance of anisotropy increases substantially, purely due to the number of directions in which anisotropy can occur. To demonstrate this link, we simulated an experiment where a material is subjected to a varying thermal history. That is, the experiment consists of repeatedly changing the temperature, and taking measurements along this time-series of different temperatures. The temperature at each time step can be thought of as one of the dimensions of the parameter space. The full set of possible applied thermal histories thus become points in the high-dimensional parameter space of temperatures.

In particular, we consider the ordering of a block copolymer, which is a self-assembling material that spontaneously organizes into a well-defined morphology when thermally annealed^37^. The material organizes into a defined unit cell locally, with ordered grains subsequently growing in size as defects annihilate^38^. We use a simple model to describe this grain coarsening process, where the grain size $\xi$ increases with time according to a power-law

19 $$\begin{aligned} \xi = k t^{\alpha } , \end{aligned}$$

where $\alpha$ is a scaling exponent (set to 0.2 for our simulations) and the prefactor *k* captures the temperature-dependent kinetics

20 $$\begin{aligned} k = A e^{-E_a/k_B T} . \end{aligned}$$

Here, $E_a$ is an activation energy for coarsening (we select a typical value of $E_a = 100 \, \mathrm {kJ/mol}$), and the prefactor *A* sets the overall scale of the kinetics (set to $3 \times 10^{11} \, \mathrm {nm}/\mathrm {s}^{\alpha }$). From these equations we construct an instantaneous growth-rate of the form:

21 $$\begin{aligned} \frac{\mathrm {d}\xi }{\mathrm {d}t} = k^{1/\alpha } \xi ^{1-1/\alpha } . \end{aligned}$$

Block copolymers are known to have an order-disorder transition temperature ($T_{\mathrm {ODT}}$) above which thermal energy overcomes the material’s segregation strength, and thus the nanoscale morphology disappears in favor of a homogeneous disordered phase. Heating beyond $T_{\mathrm {ODT}}$ thus implies driving $\xi$ to zero. We describe this ‘grain dissolution’ process using an ad-hoc form of:

22 $$\begin{aligned} \frac{\mathrm {d}\xi }{\mathrm {d}t} = - k_{\mathrm {diss}} (T-T_{\mathrm {ODT}}) , \end{aligned}$$

where we set $k_{\mathrm {diss}} = 1.0 \, \mathrm {nm\,s^{-1}\,K^{-1}}$ and $T_{\mathrm {ODT}} = 350 \, ^{\circ }\mathrm {C}$. We also apply ad-hoc suppression of kinetics near $T_{\mathrm {ODT}}$ and when grain sizes are very large to account for experimentally-observed effects. Overall, this simple model describes a system wherein grains coarsen with time and temperature, but shrink in size if the temperature is raised too high. The parameter space defined by a sequence of temperatures will thus exhibit regions of high or low grain size depending on the thermal history described by that point; moreover, there is a non-trivial coupling between these parameters since the grain size obtained for a given step of the annealing (i.e. a given direction in the parameter space) sets the starting-point for coarsening in the next step (i.e. the next direction of the parameter space).

**Figure 7:**
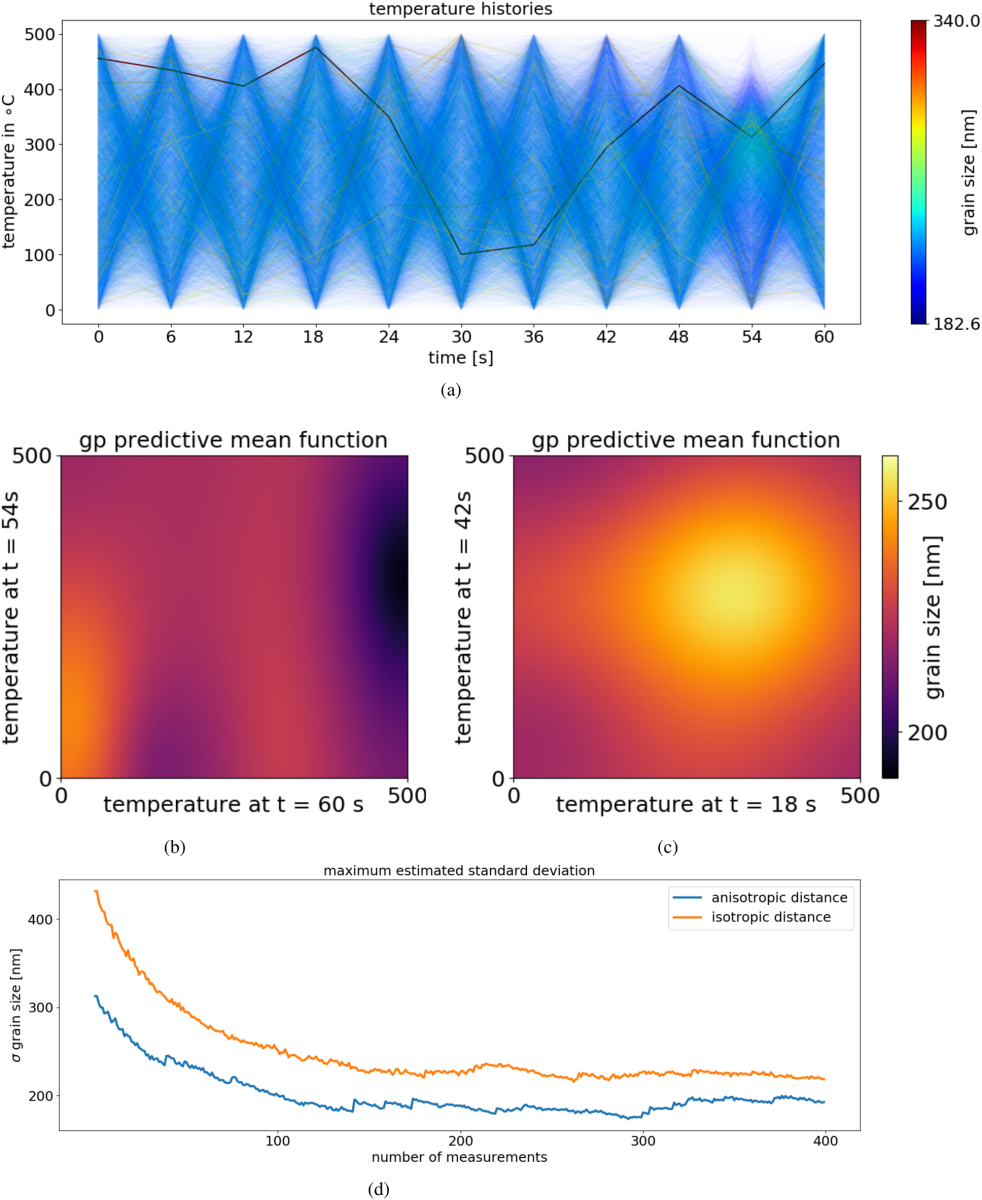
Visualization of the grain size as a function of temperature history for a simple model of block copolymer grain size coarsening. The figure demonstrates that when describing physical systems in high-dimensional spaces, strong anisotropy is frequently observed; only by taking this into account when estimating errors, will experimental guidance be optimal. (**a**) 10,000 simulated temperature histories and their corresponding grain size represented by color. The majority of histories terminate in a small grain size (blue lines). A small select set of histories yield large grain sizes (dark red lines). (**b**) Example two-dimensional slice through the 11-dimensional parameter space. The anisotropy is clearly visible. (**c**) A different two-dimensional slice with no significant anisotropy present. (**d**) The estimated maximum standard deviation across the 11-dimensional domain as function of the number of measurements during a synthetic autonomous experiment.

We select thermal histories consisting of 11 temperature selections (temperature is updated every $6 \, \mathrm {s}$), which thus defines an 11-dimensional parameter space for exploration. Each temperature history defines a point (${\mathbf{x}}~\in ~{\mathscr {X}}$) within the 11-dimensional input space. As can be seen in Fig. 7a, the majority of thermal histories terminate in a relatively small grain size (blue lines in Fig. 7a). This can be easily understood since a randomly-selected annealing protocol will use temperatures that are either too low (slow coarsening) or too high ($T>T_{\mathrm {ODT}}$ drives into disordered state). Only a subset of possible histories terminate with a large grain size (dark, less transparent lines in Fig. 7a), corresponding to the judicious choice of annealing history that uses large temperatures without crossing ODT. While this conclusion is obvious in retrospect, in the exploration of a new material system (e.g. for which the value of material properties like $T_{\mathrm {ODT}}$ are not known), identifying such trends is non-trivial. Representative slices through the 11-dimensional parameter space (Fig. 7b, c) further emphasize the complexity of the search problem, especially emphasizing the anisotropy of the problem. That is, different steps in the annealing protocol have different effects on coarsening; correspondingly the different directions in the parameter space have different characteristic length scales that must be correctly modeled (even though every direction is conceptually similar in that it describes a $6 \, \mathrm {s}$ thermal annealing process).

Autonomous exploration of this parameter space enables the construction of a model for this coarsening process. Moreover, the inclusion of anisotropy markedly improves the search efficiency, reducing the model error more rapidly than when using a simpler isotropic kernel (Fig. 7d). As the dimensionality of the problem and the complexity of the physical model increase, the utility of including an anisotropic kernel increases further still.

## Autonomous SAXS exploration of nanoscale ordering in a flow-coated polymer-grafted nanorod film

The proposed GP-driven decision-making algorithm that takes into account non-i.i.d. observation noise and anisotropy has been used successfully in autonomous synchrotron experiments. Here we present, as an illustrative example, the results of an autonomous x-ray scattering experiment on a polymer-grafted gold nanorod thin film, where a combinatorial sample library was used to explore the effects of film fabrication parameters on a self-assembled nanoscale structure.

Unlike traditional short ligand coated particles, polymer-grafted nanoparticles (PGNs) are stabilized by high molecular weight polymers at relatively low grafting densities. As a result, PGNs behave as soft colloids, possessing the favorable processing behavior of polymer systems while still retaining the ability to pack into ordered assemblies^39^. Although this makes PGNs well suited to traditional approaches for thin-film fabrication, the nanoscale assembly of these materials is inherently complex, depending on a number of variables including, but not limited to, particle-particle interactions, particle-substrate interactions, and process methodology.

The combinatorial PGN film sample was fabricated at the Air Force Research Laboratory. A flow-coating method^39^ was used to deposit a thin PGN film on a surface-treated substrate where gradients in coating velocity and substrate surface energy were imposed along two orthogonal directions over the film surface. A 250 nM toluene solution of 53 kDa polystyrene-grafted gold nanorods (94% polystyrene by volume), with nanorod dimensions of $70 \pm 6$ nm in length and $11.0 \pm 0.9$ nm in diameter (based on TEM analysis), was cast onto a functionalized glass coverslip using a motorized coating blade. The resulting film covered a rectangular area of dimensions 50 mm $\times$ 60 mm. The surface energy gradient on the glass coverslip was generated through the vapor deposition of phenylsilane^40^. The substrate surface energy varied linearly along the *x* direction from 30.5 mN/m (hydrophobic) at one edge of the film ($x=0$) to 70.2 mN/m (hydrophilic) at the other edge ($x=50$ mm). Along the *y* direction, the film-casting speed increased from 0 mm/s (at $y=0$) to 0.5 mm/s ($y=60$ mm) at a constant acceleration of 0.002 mm/s$^{2}$. The film-casting condition corresponds to the evaporative regime where solvent evaporation occurs at similar timescales to that of solid film formation^41^. In this regime, solvent evaporation at the meniscus induces a convective flow, driving the PGNs to concentrate and assemble at the contact line. The film thickness decreased with increasing coating speed, resulting in transitions from multilayers through a monolayer to a sub-monolayer with increasing *y*. This was verified by optical microscopy observations of the boundaries between multilayer, bilayer, monolayer and sub-monolayer regions, the last of which were identified by the presence of holes in the film, typically 1 $\mu$m or greater as seen in the optical images.

The objective of the autonomous synchrotron x-ray scattering experiment was two-fold, corresponding to a combination of exploration and exploitation. The first aim was to explore the dependence of the nanoscale order of the PGN film on the two fabrication parameters, i.e., the substrate surface energy and the film coating speed, or equivalently on the surface coordinates (*x*, *y*), respectively. The second aim was to exploit the knowledge gained from the exploration to locate and home in on the regions in the two-dimensional parameter space that resulted in the highest degrees of order.

The autonomous small-angle x-ray scattering (SAXS) experiment was performed at the Complex Materials Scattering (11-BM CMS) beamline at the National Synchrotron Light Source II (NSLS-II), Brookhaven National Laboratory. As described previously^17,42^, experimental control was coordinated by combining three Python software processes: *bluesky*^43^ for automated sample translations and data collection, *SciAnalysis*^44^ for real-time analysis of newly collected SAXS images, and the above GPR-based optimization algorithms for decision-making. The incident x-ray beam was set to a wavelength of 0.918 Å  (13.5 keV x-ray energy) and a size of 0.2 mm $\times$ 0.2 mm. The PGN film-coated substrate was mounted normal to the incident x-ray beam, on a set of motorized *xy* translation stages. Transmission SAXS patterns were collected on an area detector (DECTRIS Pilatus 2M) located at a distance of 5.1 m downstream of the sample, with an exposure time of 10 s/image. The SAXS results indicate that the polymer grafted nanorods tend to form ordered domains in which the nanorods lie flat and parallel to the surface and align with their neighbors. The fitting of SAXS intensity profiles via real-time analysis allowed for the extraction of quantities such as the scattering-vector position *q* for the diffraction peak corresponding to the in-plane inter-nanorod spacing $d = 2\pi /q$; the degree of anisotropy $\eta \in [0, 1]$ for the in-plane inter-nanorod alignment, where $\eta = 0$ for random orientations and $\eta = 1$ for perfect alignments^45^; the azimuthal angle $\chi$ or the factor $\cos (2\chi )$ for the in-plane orientation of the inter-nanorod alignment; and the grain size $\xi$ of the nanoscale ordered domains, which is inversely proportional to the diffraction peak width and provides a measure of the extent of in-plane positional correlations between aligned nanorods. The analysis-derived best-fit values and associated variances for these parameters were passed to the GPR decision algorithms.

**Figure 8:**
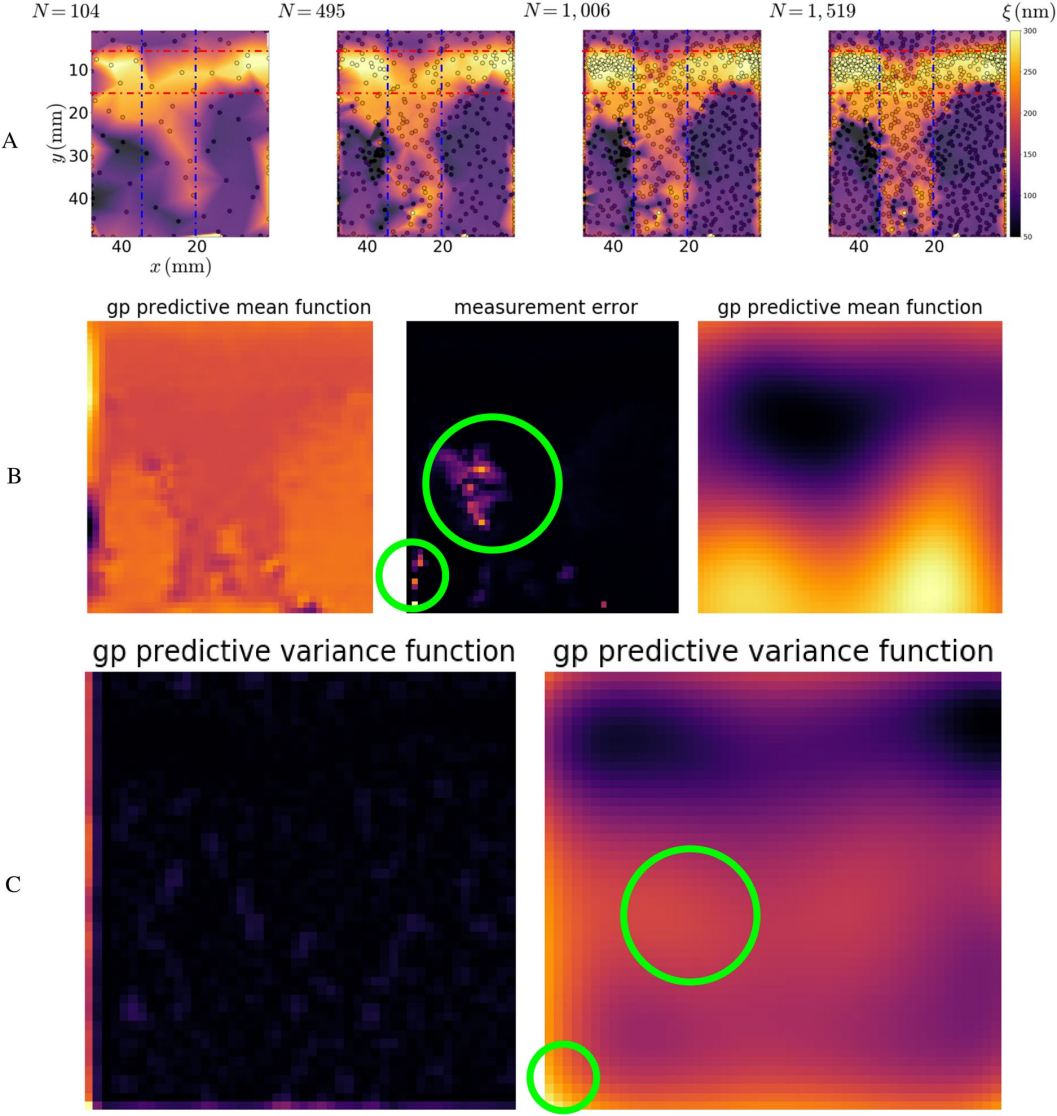
(top row, A) Results of an autonomous SAXS experiment probing the distribution of grain size ($\xi$) in a combinatorial nanocomposite sample, as a function of coordinates $(x,\,y)$ representing a two-dimensional sample-processing parameter space, for an increasing number of measurements (*N*). The sample consisted of a flow-coated film of polymer-grafted nano-rods on a surface-treated substrate, where the substrate surface energy increased linearly from $30.5 \, \mathrm {mN/m}$ (hydrophobic) at $x = 0$ to $70.2 \, \text {mN/m}$ (hydrophilic) at $x \approx 50 \, \mathrm {mm}$, and the coating speed increased at constant acceleration ($0.002 \, \text {mm/s}^{2}$) from $0 \, \text {mm/s}$ (thicker film) at $y = 0$ to $0.45 \, \text {mm/s}$ (thinner film) at $y \approx 50 \, \mathrm {mm}$. The autonomous experiment successfully identified a well-ordered region (between red lines) that corresponded to uniform monolayer domains. Blue lines mark the region of solution-meniscus instability (see text). The points show the locations of measured data points; the same axes and orientation are used in subsequent plots in this figure. (middle, row B, from the left) An exact Gaussian-process interpolation of the complete measured data-set for the peak position *q*. The data is corrupted by measurement errors that corrupt the model if standard, exact interpolation techniques are used (including GPR). The green circles mark the regions of the largest variances in the model and the corresponding high errors (measurement variances) that were recorded during the experiment. On the right is the Gaussian process model of *q*, taking into account the non-i.i.d. measurement variances. This model does not show any of the artifacts that are visible in the exact GPR interpolation. (bottom row, C) The final objective functions for no noise and non-i.i.d. noise in *q* which has to be maximized to determine the next optimal measurement. If the experiment had been steered using the posterior variances in *q* without accounting for non-i.i.d. observation noise, the autonomous experiments would have been misled significantly.

In the autonomous experiment, three analysis-derived quantities $\xi$, $\eta$, and $\cos (2\chi )$ were used as the input signals utilized by the GPR algorithms to steer the SAXS measurements as a function of surface coordinates (*x*, *y*). For the GPR computations, the search space was restricted to $1.0 \le x \le 48.0$ mm and $1.0 \le y \le 49.0$ mm. The objective function used was described previously, given by Eq. (11) of Ref.^42^. The objective function is therefore of the upper-confidence kind as described in^46^, with varying trade-off coefficient throughout the experiment. For this experiment, we used the first-order-differentiability Matérn kernel. Setting up the parameter space, or search space, has to be done initially by the user; afterward the experiment runs autonomously without human interference. For the initial part of the experiment, $N < 464$ (first 4 h), where *N* is the number of measurements completed up to a given point in the experiment, the autonomous steering utilized the exploration mode based on model uncertainty maxima^42^ for $\xi$, $\eta$, and $\cos (2\chi )$. For the later part of the experiment ($464 \le N \le 1520$ or next 11 h), the feature maximization mode^42^ was used for $\eta$, while keeping $\xi$ and $\cos (2\chi )$ in the exploration mode. We found that the nanorods in the ordered domains tended to orient such that their long axes were aligned along the *x* direction [$\cos (2\chi ) \approx 1$], i.e., perpendicular to the coating direction, and that $\xi$ and $\eta$ are strongly coupled. Figure 8A (top panels) show the *N*-dependent evolution of the model for the grain size distribution $\xi$ over the film surface. It should be noted that the entire experiment took 15 h, and that the GPR-based autonomous algorithms identified the highly ordered regions in the band $5< y < 15$ mm (between red lines in Fig. 8A), corresponding to the uniform monolayer region, within the first few hours. By contrast, grid-based scanning-probe transmission SAXS measurements would not be able to identify large regions of interest at these resolutions in such a short amount of time^17^.

The collected data is corrupted by non-i.i.d. measurement noise. While all signals are corrupted by noise, we draw attention to the peak position *q* because it shows the most obvious correlation of non-i.i.d. measurement noise and model certainty. The green circles in Fig. 8B (middle panel) and C (right panel) highlight the areas where the measurement noise affects the Gaussian-process predictive variance significantly. Note that we have not used *q* for steering in this case, but the general principle we want to show remains unchanged across all experiment results. Figure 8A shows the time evolution of the exploration of the model and the impact of non-i.i.d. noise on the model but also on the uncertainty. If *q* had been used for steering without taking into account non-i.i.d.noise into the analysis, the autonomous experiment would have been misled because predictive uncertainty due to high noise levels would not have been taken into account. Figure 8 shows that the next suggested measurement strongly depends on the noise. We want to remind the reader at this point that the next optimal measurement happens at the maximum of the GP predictive variance. The locations of the optima (Fig. 8C) are clearly different when non-i.i.d. noise is taken into account. The objective function without measurement noise (Fig. 8C, left panel) shows no preference for regions of high noise (green circles in Fig. 8B, middle panel), where preference means higher function values of the GP predictive variance. In contrast, the variance function that takes measurement noise into account (Fig. 8C, right panel) gives preference to regions (green circles) where measurement noise of the data is high. This is a significant advantage and can only be accomplished by taking into account non-i.i.d. measurement noise. In conclusion, the model that assumes no noise looks better resolved, which communicates a wrong level of confidence and misguides the steering. The model that takes into account non-i.i.d. noise finds the correct, most likely model, and the corresponding uncertainty. The algorithm also took advantage of anisotropy by learning a slightly longer length scale in the x-direction which increased the overall model certainty. Note that the algorithm used an objective function formulation that put emphasis on high-amplitude regions of the parameter space. This led to a higher resolution in those areas of interest.

The above autonomous SAXS experiment revealed interesting features from the material fabrication perspective as well. First, a somewhat surprising result is that the grain size is not observed to change significantly with surface energy (Fig. 8A). Previous work on the assembly of polystyrene-grafted spherical gold nanoparticles^39^ demonstrated a significant decrease in nanoparticle ordering when fabricating films on lower surface energy substrates (greater polymer-substrate interactions). Although the surface energies used in this study are similar, a different silane was used to modify the glass surface (phenylsilane vs octyltrichlorosilane) which may differ in its interaction with polystyrene. We also note that PGN-substrate interactions will be sensitive to the molecular orientation of the functional groups, which is known to be highly dependent on the functionalization procedure^40^. Second, an unexpected well-ordered band was identified at $20< x < 35$ mm and $y > 15$ mm (between blue lines in Fig. 8A), corresponding to the sub-monolayer region with an intermediate surface-energy range. We believe that this effect arises from instabilities associated with the solution meniscus near the middle of the coating blade ($x \sim 25$ mm). Rapid solvent evaporation often leads to undesirable effects including the generation of surface tension gradients, Marangoni flows, and subsequent contact line instabilities. This can result in the formation of non-uniform morphologies as demonstrated by the irregular region of larger grain size centered in the middle of the film and spanning the entire velocity range. Further investigations into these issues are currently in progress.

## Discussion and conclusion

In this paper, we have demonstrated the importance of including inhomogeneous (i.e. non-i.i.d.) observation noise and anisotropy into Gaussian-process-driven autonomous materials-discovery experiments.

It is very common in the scientific community to rely on Gaussian processes that ignore measurement noise or only include homogeneous noise, i.e. noise that is constant across measurements. In experimental sciences, and especially in experimental material sciences, strong inhomogeneity in measurement noise can be present and only accounting for homogeneous (i.i.d) measurement noise is therefore insufficient and leads to inaccurate models and, in the worst case, wrong interpretations and missed scientific discoveries. We have shown that it is straightforward to include non-i.i.d noise into the steering and modeling process. Figure 5 undoubtedly shows the benefit of including non-i.i.d measurement noise into the Gaussian process analysis. Figure 6 supports the conclusion we drew from Fig. 5 visually, by showing a faster error decline.

The case for allowing anisotropy in the input space can be made when there is a reason to believe that data varies much more strongly in certain directions than in others. This is often the case when the directions have different physical meanings. For instance, one direction can mean temperature, while another one can define a physical distance. In this case, accounting for anisotropy can be vastly beneficial, since the Gaussian process will learn the different length scales and use them to lower the overall uncertainty. Figure 7 shows how common anisotropy is, even in cases where it would normally not be expected, and how including it decreases the approximated error of the Gaussian process posterior mean. In our example, all axes carry the unit of temperature; even so, anisotropy is present, and accounting for it has a significant impact on the approximation error.

In our autonomous synchrotron x-ray experiment, we have seen how misleading the no-measurement-noise assumption can be. While the Gaussian process posterior mean, assuming no noise, is much more detailed in Fig. 8, it is not supported by the data which is subject to non-i.i.d. noise. In addition, we have seen that the steering actually accounts for the measurement noise if included, which leads to much a smarter decision algorithm that knows where data is of poor quality and has to be substantiated. We showed that without accounting for non-i.i.d. noise this phenomenon would not arise. We would therefore place measurements sub-optimally, wasting device access, staff time, and other resources.

It is important to discuss the computational costs that come with accounting for non-i.i.d. noise and anisotropy. While non-i.i.d. noise can be included at no additional computational costs, anisotropy potentially comes at a price. The more complex the anisotropy, the more hyper parameters have to be found. The number of hyper parameters translates directly into the dimensionality of the space over which the likelihood is defined. The training process to find the hyper parameters will therefore take longer when more hyper parameters have to be found. However, the cost per function evaluation will not change significantly. Therefore, instead of avoiding the valuable anisotropy, we should make use of modern, efficient optimization methods.

During the experiment process, the GP-based autonomous experiment keeps track of the posterior variance function. This function serves as validation for the scientists and can be used to confidently terminate the process when an uncertainty threshold is reached. Another quantity that is available to the scientist for verification and validation, is the change in differential entropy as data is collected.

While our results have shown that accounting for non-i.i.d. noise and anisotropy is highly valuable for the efficiency of an autonomously steered experiment, we have only scratched the surface of possibilities. Both proposed improvements can be seen as part of a larger theme commonly referred to as kernel design. The possibilities for improvements and tailoring of Gaussian-process-driven steering of experiments are vast. Well-designed kernels have the power to extract sub-spaces of the Hilbert space of functions, which means that constraints can be placed on the functions we consider as our model. We will look into the impact of advanced kernel designs on autonomous data acquisition in the near future.
